# Pre−treatment cytokines plus TPSA predict biochemical progression−free survival in prostate cancer metastasis and discriminate metastatic status: a retrospective study

**DOI:** 10.3389/fimmu.2025.1686570

**Published:** 2025-11-27

**Authors:** Ke-Fan Mao, Jing Sun, Chen Sun, Bo-Bo Sun, Zhi-Gang Wu, Jian Cai, Chao-Feng Zhou

**Affiliations:** 1 Department Of Urology and Andrology, The First Affiliated Hospital of Wenzhou Medical University, Wenzhou, China; 2 Wenzhou Medical University, Wenzhou, China

**Keywords:** prostate cancer, metastasis, cytokines, serum biomarkers, chemotherapy response, predictive prognostic modeling

## Abstract

**Introduction:**

Prostate cancer (PCa) ranks as the second most common malignancy in men worldwide. While serum prostate-specific antigen (PSA) is routinely monitored, its low specificity frequently leads to overdiagnosis. Cytokines within the tumor microenvironment (TME) demonstrate strong tumor-progression associations, but their combined predictive utility with PSA for metastasis and chemotherapy response remains undetermined. This study aimed to quantify cross-sectional differences in pre-treatment cytokine levels based on metastatic status, assess their prognostic value for biochemical progression-free survival in metastatic patients, and characterize cytokine profiles from baseline to biochemical recurrence.

**Methods:**

We retrospectively analyzed 328 PCa patients (175 metastatic, 153 non-metastatic), collecting data on age, smoking history, Gleason score, total PSA (TPSA), and cytokines. Metastasis-associated factors were identified by Spearman correlation and logistic regression. Prognostic models were evaluated using ROC curves/AUC analysis. Multi index combination was used to find the best prognostic group.Survival analysis employed Kaplan-Meier methodology, while Cox regression assessed post-chemotherapy PSA rebound predictors.

**Introduction:**

We find that smoking, TPSA, and IL-8 emerged as independent metastasis risk factors. Prognostic indices PRE1 (smoking, TPSA, IL-8) and PRE2 (all significant factors) achieved AUCs of 0.788 and 0.787 respectively, with PRE1 demonstrating superior calibration. The AUC of TPSA+IL-6+IL-8+IL-10 four factor combination was 0.753, and this combination yielded high prognostic performance, and the proportion of metastasis group was significantly higher than that of non-metastasis group. Univariate Cox analysis associated age, TPSA, IL-6, IL-8, and TNF-α with PSA rebound, though multivariate analysis identified no independent predictors.

**Discussion:**

These results underscore the immunological relevance of specific cytokines in PCa progression and their potential as complementary biomarkers to PSA for improving risk stratification.

## 1 Introduction

According to GLOBOCAN 2022 data from the International Agency for Research on Cancer (IARC), prostate cancer ranks as the second most frequently diagnosed malignancy and the fifth leading cause of cancer-related death among men worldwide (1). Prostate-specific antigen (PSA) is widely used to screen for and monitor prostate disease because it is highly sensitive, but it is not very specific (2, 3). Levels rise with age, infection, certain drugs, and larger glands, often producing false positives and prompting unnecessary treatment (4–7). This limits PSA’s ability to predict clinically significant cancer. Consequently, identifying novel biomarkers that complement PSA to enhance diagnostic accuracy remains critically important.

The tumor microenvironment (TME) is the immediate surroundings of cancer cells (8). It contains both cellular and non-cellular elements (9). Cellular partners include tumor-infiltrating lymphocytes, regulatory T cells, tumor-associated macrophages, cancer-associated fibroblasts, and endothelial cells; The non-cellular fraction comprises the extracellular matrix along with cytokines, chemokines, and growth factors (10, 11). Research suggests that tumor cells, immune cells, and stromal cells within the TME collectively secrete cytokines. These cytokines play a important role in the TME by modulating immune responses and influencing various facets of tumor biology. They help maintain the delicate balance between immune surveillance and immune evasion, while also regulating key processes such as tumor proliferation, survival, invasion, and metastasis (12). IL-6 is a key pro-inflammatory cytokine produced by tumor cells, fibroblasts, and immune cells. It triggers signaling cascades, including the JAK/STAT3 pathway, via the IL-6R/gp130 receptor complex, which supports tumor cell survival, proliferation, migration, and invasion. This signaling upregulates anti-apoptotic genes (e.g., Bcl-2 family members), downregulates pro-apoptotic genes, and enhances the expression of immunosuppressive molecules such as PD-L1, IDO, and IL-10, collectively promoting immune evasion. IL-6 also contributes to angiogenesis, cancer-associated fibroblast (CAF) activation, extracellular matrix remodeling, immune cell infiltration, and metabolic reprogramming. For instance, IL-6 stimulates VEGF secretion to foster angiogenesis and activates CAFs to produce matrix metalloproteinases (MMPs) and collagenases, which degrade the basement membrane and facilitate tumor cell migration (13). In prostate cancer, IL-6 drives biochemical alterations—such as upregulation of survival genes, suppression of apoptotic genes, and increased invasiveness—and is linked to poor clinical outcomes (14).

IL-8 enhances prostate cancer cell proliferation, migration, invasion, epithelial–mesenchymal transition (EMT), and angiogenesis by signaling through CXCR1/2 receptors. The IL-8/CXCR1/2 axis promotes the accumulation of tumor-associated neutrophils, macrophages, and myeloid-derived suppressor cells (MDSCs) within the tumor microenvironment (TME), thereby amplifying immunosuppression (15). In castration-resistant and metastatic prostate cancer, IL-8 expression is elevated and correlates with therapeutic resistance and heightened invasiveness. Moreover, prostate cancer-derived exosomes can deliver IL-8, impairing CD8+ T cell function and facilitating tumor metastasis and immune escape (16).

TNF-α is mainly secreted by immune cells such as macrophages and T cells. It activates NF-κB and MAPK signaling pathways to suppress apoptosis, promote proliferation and survival, and induce EMT—mediated by transcription factors including Snail and ZEB1—thereby enhancing the migratory, invasive, and metastatic capacity of prostate cancer cells. TNF-α also stimulates vascular endothelial growth factor (VEGF) secretion, supporting tumor growth and metastasis, while recruiting MDSCs and regulatory T cells (Tregs) and promoting M2 macrophage polarization. These actions collectively impair effector immune cell function and foster an immunosuppressive TME (17). Evidence suggests that TNF-α signaling contributes to resistance against chemotherapy and endocrine therapies in prostate cancer. Its downstream effectors, such as NF-κB, interact with androgen receptor signaling, enabling tumor cell survival and proliferation even under androgen-deprived conditions, thereby advancing the development of castration-resistant prostate cancer (18).

Given PSA’s limitations and the functional significance of cytokines, this study investigates the clinical value of combining serum PSA with cytokine profiling in prostate cancer patients. We will examine how these biomarkers relate to the onset, growth, spread, and outcome of prostate cancer, test whether their combined use improves prediction of disease progression, and assess whether cytokine levels forecast a post-chemotherapy rise in PSA. It is hoped that our research can provide new ideas for the prediction and monitoring of the development of prostate cancer.

## 2 Materials and methods

### 2.1 Patients and data collection

This retrospective study received approval from the Ethics Committee of The First Affiliated Hospital of Wenzhou Medical University (No: KY2025-R224), with ethics approval reference, and was conducted in accordance with the World Medical Association Declaration of Helsinki. Written informed consent was obtained from all participants. We retrospectively analyzed medical records from 2023 to 2025, enrolling 328 eligible prostate cancer patients (153 non-metastatic, mean age 70.83 years; 175 metastatic, mean age 72.69 years). All non-metastatic patients are in the TII stage, while all metastatic patients are in the TIV stage because they have pelvic lymphnode or bone metastases. All patients received standard androgen deprivation therapy (ADT) and a proportion of those in the metastatic group also received docetaxel-based chemotherapy as part of their treatment regimen. All patients did not receive surgical treatment during cytokine testing and all diagnoses were histologically confirmed through ultrasound-guided transperineally prostate biopsy. Metastasis status was independently evaluated by two radiologists using MRI and/or PET-CT. To ensure data reliability, we excluded patients with comorbidities including acute/chronic inflammation, additional malignancies, autoimmune disorders, major organ dysfunction, infectious diseases, or severe neuropsychiatric conditions. Collected baseline data comprised age, smoking history and Gleason score, and collect TPSA and cytokine data in blood tests of patients. The levels of five cytokines (IL-6, IL-8, IL-10, IL-1β, and TNF-α) were assessed by ELISA at distinct clinical timepoints: at the initial cancer diagnosis for non-metastatic patients and upon the initial radiological detection of metastasis for those in the metastatic group. The complete list of cytokines (including detection methods, reference ranges, and reasons for selection) is shown in **Supplementary Table S1**.

**Figure 1:**
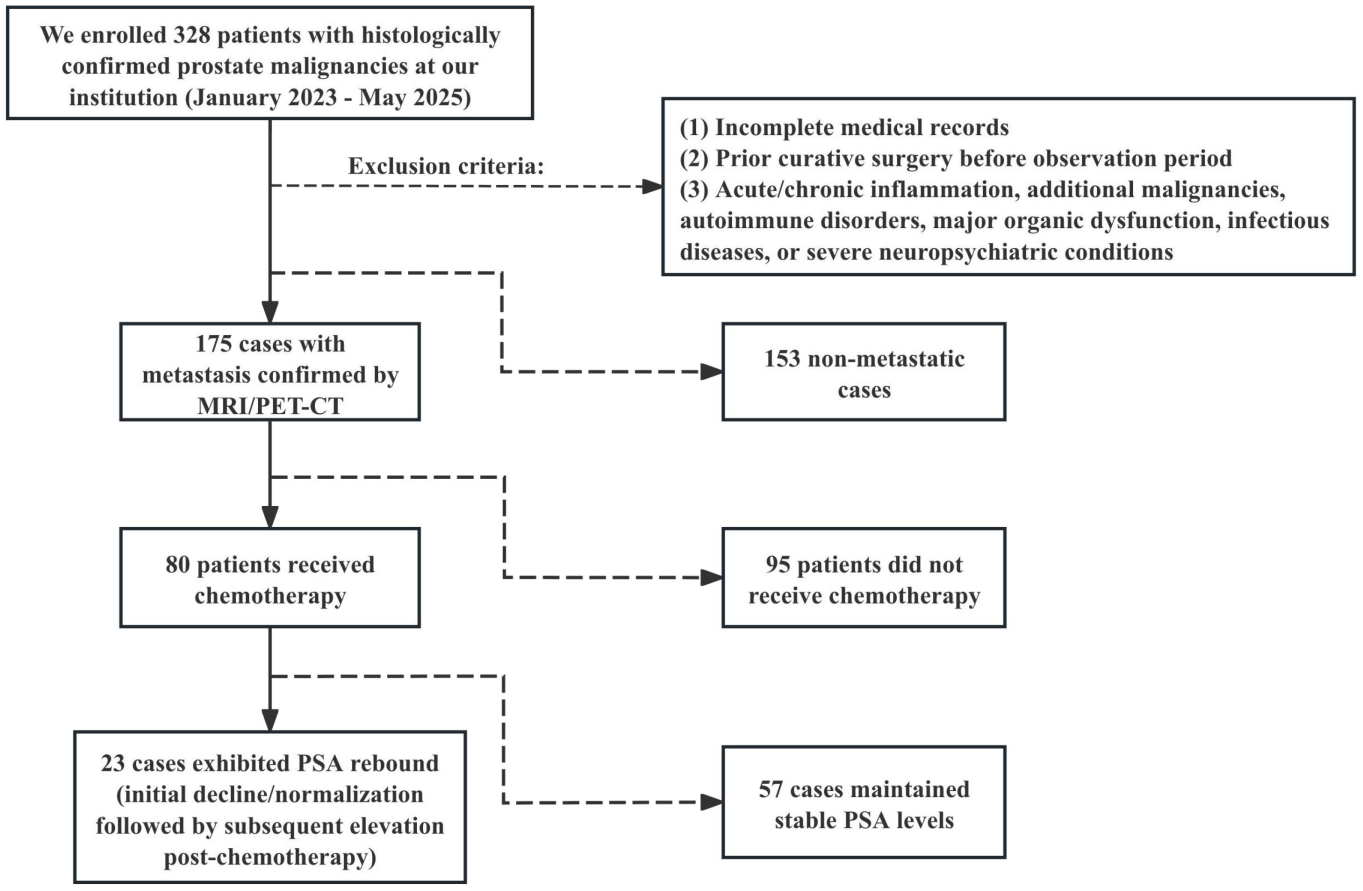
Research flowchart. Between January 2023 and May 2025, a total of 328 patients diagnosed with prostate malignancy after prostate biopsy were screened.

Among metastatic patients, 80 received chemotherapy (mean age 70.65 years), with 23 developing post-chemotherapy TPSA rebound (mean age 72.22 years). TPSA rebound is defined as the re-elevation of TPSA levels following chemotherapy, in the absence of radiographic evidence of disease progression. Therapeutic response was defined as time from chemotherapy initiation to TPSA rebound or last follow-up for non-rebound cases. **Figure 1** presents the study flowchart.

### 2.2 Statistical analysis

All analyses were conducted using SPSS version 27.0 (IBM Corp., Armonk, NY, USA) and R version 4.5.0 (R Foundation for Statistical Computing, Vienna, Austria). Missing data (4.8%) were imputed using SPSS Multiple Imputation Method. The “methodological sensitivity analysis” (including “Linear regression for continuous variables”, “Predictive Mean Matching (PMM)” and “Bayesian Linear Regression”) was conducted after imputation to assess the sensitivity of the results. The findings indicate that the direction of the coefficients was consistent, the conclusions regarding statistical significance remained unchanged, the differences in coefficient values were small, and there was substantial overlap in the 95% confidence intervals across all methods. This suggests that our main conclusions are not sensitive to the choice of imputation method. The comparative results are presented in **Supplementary Table S2**. Normality was assessed using Kolmogorov-Smirnov tests. Normally distributed continuous variables underwent independent t-tests, categorical variables chi-square tests, and non-parametric data Mann-Whitney U tests. Univariate and multivariate logistic regression calculated odds ratios (ORs) with 95% confidence intervals (CIs) for metastasis associations. Prognostic performance was quantified via receiver operating characteristic (ROC) curves and area under the curve (AUC) using R packages such as “rms”, “ggplot2”, “caret” and “dplyr”. Optimal cutoffs were determined for continuous variables (survminer, survival packages) prior to dichotomization. Kaplan-Meier curves with log-rank tests and Cox proportional-hazards regression identified OS-associated factors. A prognostic nomogram was developed in R. All analyses employed two-tailed tests with statistical significance defined at P < 0.05.

## 3 Results

### 3.1 Participant characteristics

Our cohort comprised 328 prostate cancer patients: 175 (53.4%) with metastasis and 153 (46.6%) without metastasis. Significant intergroup differences emerged in baseline characteristics and serum biomarker profiles. As detailed in **Table 1**, metastatic patients demonstrated statistically significant variations in age (P = 0.026), smoking history (P < 0.001), Gleason score (P < 0.001), TPSA (P < 0.001), IL-6 (P = 0.026), IL-8 (P < 0.001), IL-10 (P = 0.021), and TNF-α (P = 0.003). No significant difference was observed in IL-1β levels (P = 0.808).

**Table 1:** Comparison of baseline characteristics between metastatic and non-metastatic prostate cancer patients.

Characteristic	Metastatic	Non-metastatic	Total	P-value
Age (years)				
mean(sd)	72.7(8.4)	70.8(7.6)	71.8(8.1)	0.026
median(q1, q3)	74.0(67.0,77.0)	71.0(65.0,76.0)	72.0(66.0,77.0)	
Smoking, n(%)				
Yes	56(32.0)	33(21.6)	89(27.1)	<0.001
No	119(68.0)	120(78.4)	239(72.9)	0.768
TPSA(ng/ml)				
mean(sd)	235.7(352.9)	65.5(190.2)	156.3(100.5)	<0.001
median(q1, q3)	64.2(9.1,302.8)	8.8(4.8,23.5)	19.1(5.3,115.2)	
IL-6(pg/mL)				
mean(sd)	127.8(863.8)	97.4(596.6)	113.6(750.1)	0.026
median(q1, q3)	18.1(5.1,37.2)	28.2(6.8,52.8)	19.9(5.5,48.6)	
IL-8(pg/mL)				
mean(sd)	43.3(77.1)	25.9(62.7)	35.2(71.2)	<0.001
median(q1, q3)	16.6(6.6,43.3)	11.9(7.7,21.3)	14.0(6.9,34.6)	
IL-10(pg/mL)				
mean(sd)	6.5(9.9)	7.1(9.2)	6.8(9.6)	0.021
median(q1, q3)	3.2(2.5,5.6)	4.1(2.5,8.7)	3.8(2.5,7.5)	
IL-1β(pg/mL)				
mean(sd)	2.6(1.0)	2.9(3.6)	2.8(2.5)	0.808
median(q1, q3)	2.5(2.5,2.5)	2.5(2.5,2.5)	2.5(2.5,2.5)	
TNF-α(pg/mL)				
mean(sd)	3.1(6.1)	2.6(0.3)	2.8(4.4)	0.003
median(q1, q3)	2.5(2.5,2.5)	2.5(2.5,2.5)	2.5(2.5,2.5)	
Gleason,n(%)				
≤ 6	3(1.7)	13(8.5)	16(4.9)	<0.001
7	14(8.0)	64(41.8)	78(23.8)	<0.001
≥ 8	158(90.3)	76(49.7)	234(71.3)	<0.001

### 3.2 Analysis of metastasis-associated factors

Spearman correlation analysis of **Table 1** variables identified significant associations with metastasis for age (P = 0.026), smoking (P < 0.001), TPSA (P < 0.001), IL-6 (P = 0.026), IL-8 (P < 0.001), IL-10 (P = 0.021), TNF-α (P = 0.003), and Gleason score (P < 0.001). Subsequent univariate logistic regression (excluding Gleason score) revealed significant effects for age (OR = 1.029, 95% CI: 1.002-1.058; P = 0.038), smoking (OR = 1.041, 95% CI: 1.027-1.055;P < 0.001), TPSA (OR = 1.003, 95% CI: 1.002-1.004; P < 0.001), and IL-8 (OR = 1.007, 95% CI: 1.002-1.013; P = 0.008). Multivariate analysis confirmed smoking (OR = 1.038, 95% CI: 1.023-1.053; P < 0.001), TPSA (OR = 1.003, 95% CI: 1.001-1.004; P < 0.001), and IL-8 (OR = 1.006, 95% CI: 1.000-1.012; P = 0.039) as independent risk factors for metastasis.

**Figure 2:**
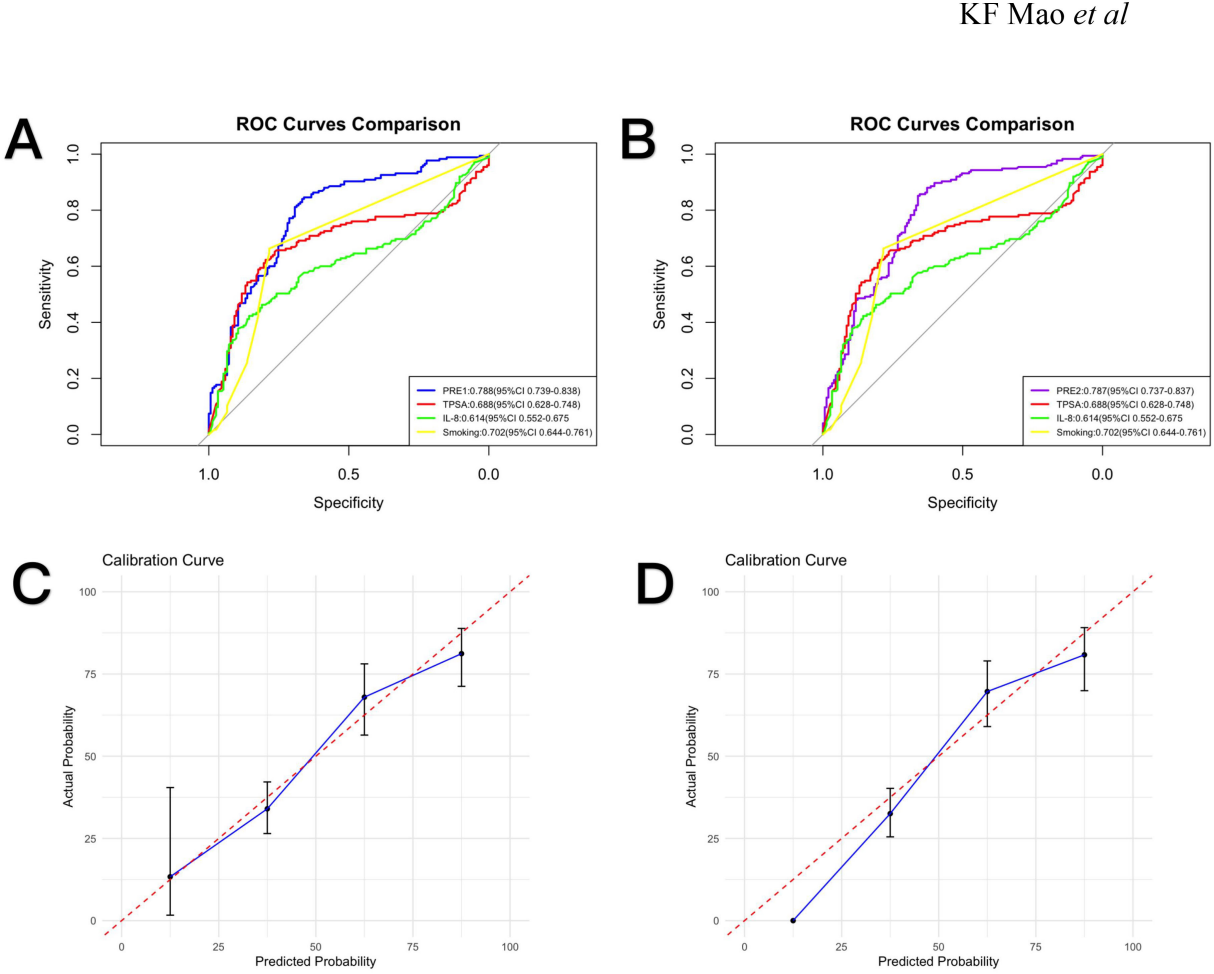
Receiver operating characteristic (ROC) curves and calibration plots. **(A)** ROC curves comparing PRE1, TPSA, IL-8, and smoking status with corresponding AUC values (95% CI). **(B)** ROC curves for PRE2, TPSA, IL-8, and smoking status with AUC values (95% CI). **(C)** Calibration curve for PRE1. **(D)** Calibration curve for PRE2.

Using multivariate logistic regression results (**Table 2**), we developed two prognostic indices: PRE1 (incorporating all Spearman-significant factors) and PRE2 (utilizing only independent risk factors: smoking, TPSA and IL-8).To evaluate diagnostic performance, we constructed receiver operating characteristic (ROC) curves and calculated area under the curve (AUC) values, comparing models against individual risk factors. PRE1 demonstrated superior performance (AUC = 0.788; **Figure 2A**) with excellent calibration (**Figure 2C**), while PRE2 showed comparable discrimination (AUC = 0.787; **Figure 2B**) but suboptimal calibration (**Figure 2D**). To compare the prognostic performance between PRE1 and PRE2, we conducted a DeLong test for their receiver operating characteristic (ROC) curves. The area under the curve (AUC) was 0.751 for PRE1 and 0.749 for PRE2, resulting in a negligible difference of 0.002. The DeLong test revealed no statistically significant difference in discriminative ability (Z = 0.222, p = 0.824), with a 95% confidence interval for the AUC difference ranging from -0.020 to 0.025.

**Table 2:** Univariate and multivariate logistic regression analysis of prostate cancer metastasis (OR: odds ratio; CI: confidence interval).

Variable	Univariate analysis		Multivariate analysis	
	OR(95%CI)	P value	OR(95%CI)	P value
Age (y)	1.029(1.002-1.058)	0.038	1.011(0.981-1.043)	0.473
Smoking	1.041(1.027-1.055)	<0.001	1.038(1.023-1.053)	<0.001
TPSA(ng/ml)	1.003(1.002-1.004)	<0.001	1.003(1.001-1.004)	<0.001
IL-6(pg/mL)	1.000(1.000-1.000)	0.689	–	
IL-8(pg/mL)	1.007(1.002-1.013)	0.013	1.006(1.000-1.012)	0.039
IL-10(pg/mL)	0.993(0.971-1.016)	0.544	–	
IL-1β(pg/mL)	0.923(0.780-1.093)	0.354	–	
TNF-α(pg/mL)	1.034(0.961-1.113)	0.365	–	

We systematically evaluated 57 serum biomarker combinations (2-6 factors) for their association with metastasis risk (**Tables 3**, **4**).

**Table 3:** Biomarker combinations and their prognostic performance (Groups a-q).

	I	II	III	IV	V	VI	VII	VIII	IX	X	XI	XII	XIII	XIV	XV	XVI	XVII
TPSA	<0.001	<0.001	<0.001	<0.001	<0.001	-	-	-	<0.001	<0.001	<0.001	-	-	<0.001	<0.001	<0.001	<0.001
IL-6	-	0.651	-	-	-	0.999	-	-	0.717	-	-	0.769	0.989	0.552	0.728	-	0.558
IL-8	0.047	0.044	0.006	0.024	0.046	0.009	0.004	0.007	0.011	0.005	0.006	0.007	0.009	0.009	0.010	0.005	0.009
IL-10	-	-	0.030	-	-	0.057	0.068	0.043	0.032	0.049	0.032	0.070	0.060	0.040	0.034	0.052	0.043
IL-1β	-	-	-	0.270	-	-	0.344	-	-	0.422	-	0.331	-	0.400	-	0.420	0.396
TNF-α	-	-	-	-	0.342	-	-	0.377	-	-	0.368	-	0.377	-	0.369	0.367	0.369
AUC	0.736	0.737	0.749	0.743	0.724	0.635	0.637	0.629	0.753	0.754	0.745	0.638	0.628	0.754	0.749	0.749	0.753

*The complete definition of each Biomarker Signature Group (a) to (q) is provided in Supplementary **Supplementary Table S3**. *

Dashes “-” indicate excluded variables; numerical values represent P-values; AUC values denote prognostic accuracy.

**Table 4:** Prevalence of biomarker signatures in metastatic versus non-metastatic cohorts.

Group	Team members	Metastatic	Non-metastatic
I, n(%)	TPSA + IL - 8	75(42.9%)	44(28.8%)
II, n(%)	TPSA + IL - 6 + L - 8	61(34.9%)	39(25.5%)
III, n(%)	TPSA + IL - 8 + L - 10	22(12.8%)	22(14.4%)
IV, n(%)	TPSA + IL - 8 + IL - 1β	0(0%)	1(0.7%)
V, n(%)	TPSA + IL - 8 + TNF-α	2(1.1%)	0(0%)
VI, n(%)	IL - 6 + IL - 8 + L - 10	27(15.4%)	30(19.6%)
VII, n(%)	IL - 8 + L - 10 + IL - 1β	0(0%)	1(0.7%)
VIII, n(%)	IL - 8 + L - 10 + TNF-α	0(0%)	0(0%)
IX, n(%)	TPSA + IL - 6 + IL - 8 + L - 10	20(11.4%)	22(14.4%)
X, n(%)	TPSA + IL - 8 + IL - 10 + IL - 1β	0(0%)	1(0.7%)
XI, n(%)	TPSA + IL - 8 + IL - 10 + TNF-α	0(0%)	0(0%)
XII, n(%)	IL - 6 + IL - 8 + IL - 10 + IL - 1β	0(0%)	1(0.7%)
XIII, n(%)	IL - 6 + IL - 8 + IL - 10 + TNF-α	0(0%)	0(0%)
XIV, n(%)	TPSA + IL - 6 + IL - 8 + IL - 10 + IL - 1β	0(0%)	1(0.7%)
XV, n(%)	TPSA + IL - 6 + IL - 8 + IL - 10 + TNF-α	0(0%)	0(0%)
XVI, n(%)	TPSA + IL - 8 + IL - 10 + IL - 1β + TNF-α	0(0%)	0(0%)
XVII, n(%)	TPSA + IL - 6 + IL - 8 + IL - 10 + IL - 1β + TNF-α	0(0%)	0(0%)

TPSA demonstrated consistent significance across combinations, confirming its strong metastasis association and central prognostic role. IL-8 showed significant contributions in multiple combinations (Group a, Group b and Group c), suggesting critical involvement in metastasis and synergistic enhancement of prognostic accuracy. IL-10, IL-1β and TNF-α exhibited context-dependent significance, indicating combinational specificity for prognostic value. Progressive AUC improvements were observed: TPSA + IL-8 (Group a: AUC = 0.736) → TPSA + IL-6 + IL-8 (Group b: AUC = 0.737) → TPSA + IL-6 + IL-8 + IL-10 (Group i: AUC = 0.753) → TPSA + IL-6 + IL-8 + IL-10 + IL-1β (Group j: AUC = 0.754), demonstrating synergistic prognostic enhancement. Six-factor combinations (Group q: AUC = 0.753) showed no additional gain, suggesting biomarker redundancy. TPSA-independent panels (e.g., Group f: IL-6 + IL-8 + IL-10, AUC = 0.635) demonstrated substantially lower prognostic capacity.

We further validated high-performance combinations by analyzing their valence in patient horses. TPSA+IL-8 positivity was significantly elevated in static patients (42.9% vs. 28.8%; **Table 4**). Similarity, TPSA+IL-6+IL-8 valence was higher in metastasis (34.9% vs. 25.5%; **Table 4**). These distribution patterns broadly align with the findings presented in **Table 3**, suggesting that these combinations hold potential as predictive tools for metastasis. However, **Table 4** only details the proportion of cases with elevated values for each combination in the metastasis and non-metastasis groups. While this information may aid in further refining the model, it offers limited insight into predictive performance, require a consideration of additional factors.

### 3.3 Analysis of post-chemotherapy PSA rebound in metastatic PCa

In the chemotherapy-treated metastatic subgroup, we analyzed biomarker associations with post-chemotherapy TPSA rebound. Univariate Cox regression (**Table 5**) identified significant associations with earlier rebound for age (HR = 2.768, 95% CI: 1.061-7.222; P = 0.031), TPSA (HR = 6.924, 95% CI: 0.925-51.820; P = 0.029), IL-6 (HR = 4.972, 95% CI: 1.130-21.880; P = 0.020), IL-8 (HR = 7.446, 95% CI: 0.968-57.258; P = 0.026), and TNF-α (HR = 3.944, 95% CI: 0.907-17.139; P = 0.049). Multivariate analysis revealed no independent prognostic factors (**Table 5**).

**Table 5:** Univariate and multivariate cox regression analysis of factors affecting chemotherapy efficacy in metastatic prostate cancer patients (n=80).

Variable	Univariate analysis		Multivariate analysis	
	HR(95%CI)	P value	HR(95%CI)	P value
Age(y)	2.768(1.061-7.222)	0.031	2.102(0.776-5.696)	0.144
Smoking	0.559(0.237-1.315)	0.180	–	
TPSA(ng/ml)	6.924(0.925-51.820)	0.029	4.335(0.544-34.537)	0.166
IL-6(pg/mL)	4.972(1.130-21.880)	0.020	2.574(0.559-11.843)	0.225
IL-8(pg/mL)	7.446(0.968-57.258)	0.026	3.087(0.374-25.466)	0.295
IL-10(pg/mL)	0.884(0.374-2.088)	0.780	–	
IL-1β(pg/mL)	3.997(0.891-17.940)	0.054	–	
TNF-α(pg/mL)	3.944(0.907-17.139)	0.049	2.302(0.510-10.385)	0.278

**Figure 3:**
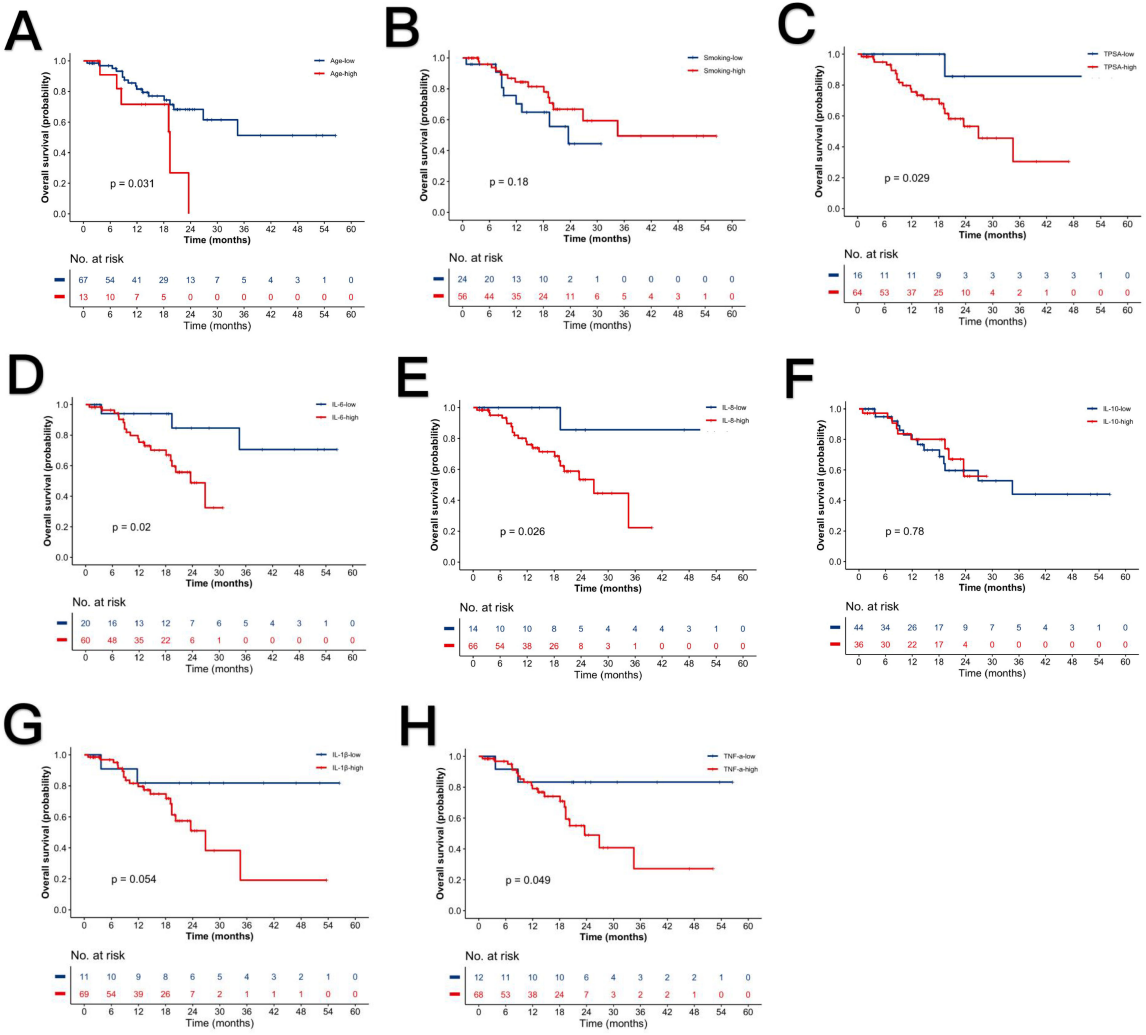
Kaplan-Meier survival curves stratified by clinical parameters. **(A)** Advanced vs. younger age. **(B)** Smokers vs. non-smokers. **(C)** High vs. low TPSA. **(D)** High vs. low IL-6. **(E)** High vs. low IL-8. **(F)** High vs. low IL-10. **(G)** High vs. low IL-1β. **(H)** High vs. low TNF-α. P-values denote log-rank test significance.

**Figure 4:**
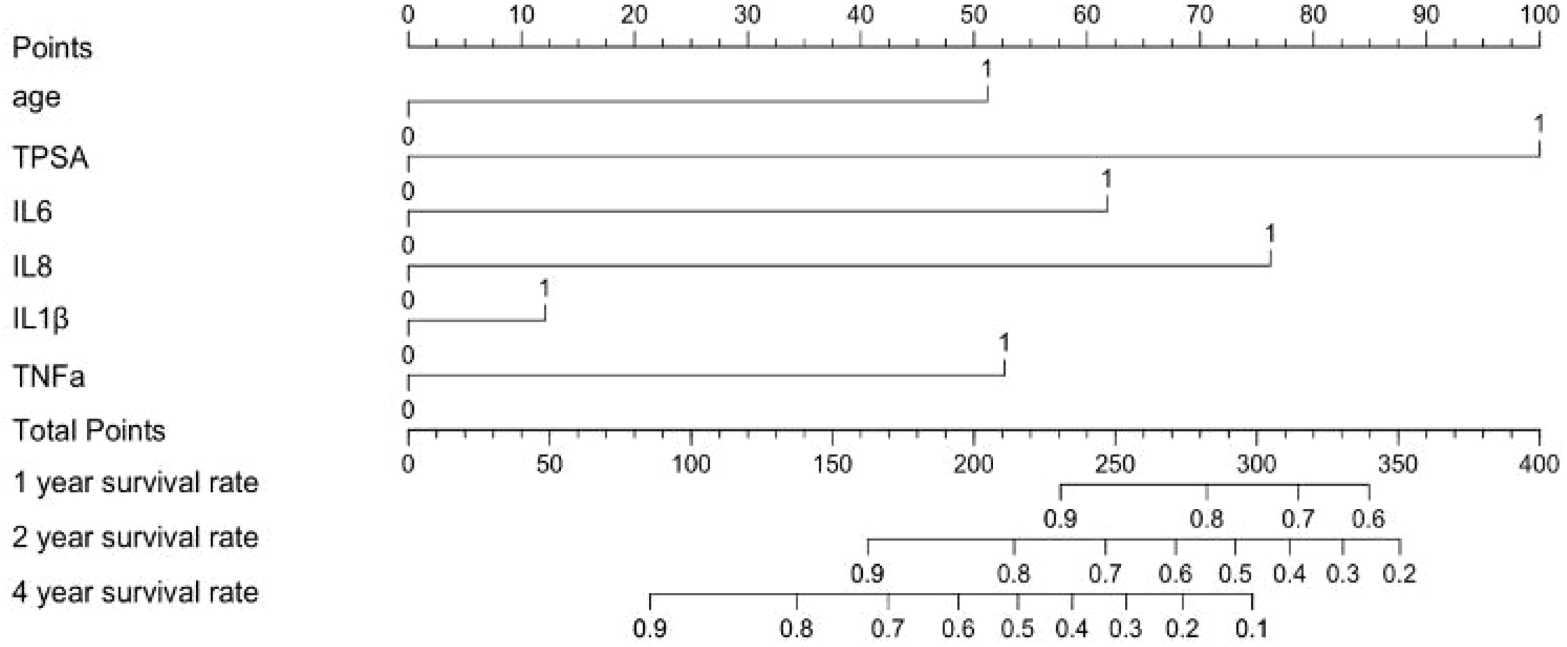
Prognostic nomogram for survival probability in prostate cancer patients. ‘1’ represents low, ‘2’ represents high.

Kaplan-Meier curves illustrate survival differences stratified by these parameters (**Figure 3**). The nomogram in **Figure 4** was constructed based on a Cox proportional hazards model, incorporating variables that reached significance (P<0.05) or a borderline level (P<0.1) in univariable analyses (age, TPSA, IL-6, IL-8, IL-1β and TNF-α), continuous biomarkers were dichotomized using the optimal cut-off identified by the Youden index in ROC analysis. The model’s regression coefficients were converted to a points scale, and the summation of these points is mapped to the predicted probabilities of survival at pre-defined time horizons. The nomogram indicates poorer outcomes with elevated TPSA and cytokine levels, consistent with univariate findings despite the absence of independent prognostic factors in multivariate analysis.

## 4 Discussion

Prostate cancer ranks as the second most diagnosed malignancy and fifth leading cause of cancer-related mortality among men globally (19). The tumor microenvironment (TME) critically regulates oncogenesis, progression, and metastasis (20). Cytokines, particularly IL-6 and IL-8, serve pivotal roles in prostate carcinogenesis, where tumor-secreted factors remodel the TME to accelerate disease progression (21, 22).

Our study included 12 cytokines, but based on the key drivers of malignancy-related cycles, we selected IL-6, IL-8, IL-10, IL-1β, and TNF-α as our primary factors for investigation. This selection was based on their central roles in the mature pro-metastatic signaling network: IL-1β activates key pathways such as NF-κB, which strongly upregulates the expression of IL-6, TNF-α, and IL-8, initiating a malignant feedback loop. The core of this loop, driven by IL-6, TNF-α, and IL-8, directly promotes tumor cell survival, proliferation, and invasion, while also inducing angiogenesis, thereby facilitating tumor metastasis. Concurrently, the immune-suppressive microenvironment shaped by this process secretes high levels of IL-10, which suppresses T cell function and antigen presentation, providing a barrier for immune evasion by tumor cells (23). Analysis of these cytokines and other risk factors, such as TPSA, revealed that smoking, elevated serum TPSA, and high IL-8 levels are independent risk factors for metastasis. The established associations between elevated TPSA, smoking history, and metastasis have been well-documented in previous studies (24–28).

Using multivariate-identified risk factors (smoking, TPSA, IL-8) for PRE1 and all significantly correlated factors (age, smoking, TPSA, IL-6, IL-8, IL-10, TNF-α) for PRE2, we developed prognostic models. PRE1 demonstrated superior calibration (AUC = 0.788) versus PRE2 (AUC = 0.787), potentially due to PRE2’s exclusion of outcome-associated variables. PRE1’s robust calibration supports combined TPSA-cytokine profiling as a promising biomarker strategy for disease progression monitoring. The DeLong test indicated no statistically significant difference in discriminatory ability between PRE1 and PRE2. Given that no significant difference in discrimination was found between PRE1 and PRE2, and given the practical value of model parsimony, we propose PRE1 as a simplified model that offers a favorable balance of prognostic accuracy and calibration. But its clinical utility should be confirmed through validation in independent, larger-scale cohorts.

To further analyze whether cytokine combined with TPSA detection can help predict prostate cancer metastasis more accurately, we examined 57 marker combinations ranging from two to six variables. We find that the group of TPSA+IL-6+IL-8+IL-10 offers the best trade-off between prognostic power and clinical clarity. Most other panels that included PSA and/or IL-8 also performed well. These results show that although TPSA often causes over diagnosis due to its low specificity, it still has a good reference value for predicting prostate cancer metastasis, and cytokines combined with TPSA can provide more accurate auxiliary diagnostic value for the assessment of prostate cancer metastasis risk than TPSA alone, but its clinical application still needs larger samples and in-depth research. Interestingly, in our study, the addition of IL-10 resulted in a decrease in the model’s AUC from 0.788 to 0.753, highlighting the dual role of IL-10 in immune responses. As an immunosuppressive cytokine, IL-10 may suppress the activity of immune cells such as T cells and macrophages, leading to reduced expression of key biomarkers and subsequently weakening the model’s diagnostic performance. Additionally, IL-10 might promote immune tolerance or interact with other immune factors, such as IL-6 and TNF-α, further compromising immune response efficacy. These findings suggest that IL-10’s effects are complex and context-dependent in different immune microenvironments (29). Future research should further investigate the mechanisms of IL-10 in various disease contexts to better understand its impact on immune diagnostics.

In a subgroup of 80 men with metastatic prostate cancer receiving chemotherapy, we simply explored how age, serum PSA, and cytokines influence the time to PSA rebound after treatment. Univariate analysis associated advanced age, elevated TPSA, IL-6, IL-8, and TNF-α with shorter rebound intervals. But multivariate analysis identified no independent predictors. This suggests that these factors interact or overlap when analyzed together. Potential explanations include: cytokine pathway interactions (IL-6 proliferation, IL-8 migration/angiogenesis, TNF-α protumor signaling), age-related inflammatory/TPSA correlations, and limited statistical power.

It should be explained that Gleason score was not included in the regression analysis model of this study. Gleason score is the core histological basis for the diagnosis, risk stratification and treatment of prostate cancer (30). About 50% of high-risk patients (≥ 8 points) have micro metastasis at the time of diagnosis, suggesting high invasiveness and metastasis tendency, which may interfere with the prediction model based on serum markers (31). The high correlation between Gleason score and prostate cancer may further lead to overfitting. In addition, Gleason score depends on invasive prostate biopsy (32). Therefore, in view of its potential strong interference and invasive access, this study chose to exclude this variable to focus on the evaluation of serum markers.

This work offers an option toward early detection of metastasis and assessment of chemotherapy response in prostate cancer, yet several limitations remain. The cohort was small and from a single center, which may introduce selection bias. Although the ROC/AUC results are statistically significant, larger prospective studies and external validation across hospitals and ethnicities are required before clinical use. PRE1 calibration may only reflect the characteristics of the training data and needs to be validated in an independent population. Multivariable Cox analysis did not show any independent predictors, validation in a larger cohort is therefore essential. However, due to the limited number of studies, the multivariable Cox regression analysis of factors associated with TPSA rebound in the chemotherapy subgroup was underpowered and carried an increased risk of overfitting. Therefore, future studies should aim to expand the sample size or employ penalized regression techniques to strengthen the analysis. Future research should test these findings in broader and well-characterized cohorts, clarify how cytokines interact and combine, create more accurate prediction tools, and evaluate individual cytokines as therapeutic targets while recalibrating models as populations and assays evolve.

## 5 Conclusion

Our study establishes that combined serum TPSA and cytokine profiling enhances metastasis prediction in prostate cancer. The PRE1 model (smoking, TPSA and IL-8) showed good prognostic performance (AUC = 0.788) and superior calibration. The combination of TPSA+IL-6+IL-8+IL-10 has excellent prediction efficiency (AUC = 0.753). These results suggest that cytokines combined with TPSA detection can provide more accurate auxiliary diagnostic value for the assessment of prostate cancer metastasis risk. In metastatic patients receiving chemotherapy, age, TPSA, IL-6, IL-8, and TNF-α showed univariate associations with PSA rebound, though multivariate analysis revealed no independent predictors, likely reflecting cytokine pathway crosstalk and variable collinearity.

## Data Availability

The original contributions presented in the study are included in the article/supplementary material. Further inquiries can be directed to the corresponding author.
