# Supplementary material for: Pre−treatment cytokines plus TPSA predict biochemical progression−free survival in prostate cancer metastasis and discriminate metastatic status: a retrospective study
Source: Front Immunol. 2025 Nov 27;16:1686570. doi: 10.3389/fimmu.2025.1686570 (PMC12695854; doi:10.3389/fimmu.2025.1686570)
Supplement: Supplementary file 1 [file Table1.docx]

**Table S1.** The complete list of cytokines.

| **Cytokine** | **Detection Method** | **Reference Range (pg/ml)** | **Brief Description** |
| --- | --- | --- | --- |
| **IL-2** | ELISA | <6.13 | effector T-cell expansion and the development of anti-tumor immunity; reflect the state of T-cell activation in the TME. |
| **IL-4** | ELISA | <4.72 | promote B-cell activation and humoral immunity; contribute to an immunosuppressive microenvironment. |
| **IL-6** | ELISA | <6.28 | pleiotropic pro-inflammatory cytokine; directly promotes cancer cell proliferation, therapy resistance, and metastatic potential; shaping an immunosuppressive tumor microenvironment and linked to PCa progression. |
| **IL-10** | ELISA | <6.09 | inhibits antigen presentation and T-cell function; associated with regulatory immune cell activity. |
| **TNF-α** | ELISA | <5.12 | promote tumor cell survival, proliferation, and angiogenesis via the NF-κB pathway. |
| **‌IFN-r**‌ | ELISA | <6.77 | enhances antigen presentation, has direct anti-proliferative effects on cancer cells. |
| **IL-17A** | ELISA | <11.17 | linking inflammation to cancer; promote tumor growth by stimulating angiogenesis and recruiting immunosuppressive myeloid cells. |
| **IL-1β** | ELISA | <9.23 | drives the production of other cytokines (e.g., IL-6, IL-8) ; a key player in chronic inflammation-driven tumorigenesis. |
| **IL-5** | ELISA | <4.90 | responsible for the growth, differentiation, and activation of eosinophils. |
| **IL-12P70** | ELISA | <4.29 | drives the differentiation of naive T cells into Th1 cells and enhances the cytotoxic activity of NK and T cells; effective anti-tumor immunity. |
| **‌IFN-α** | ELISA | <6.51 | direct anti-proliferative effects on tumor cells. |
| **IL-8** | ELISA | <15.56 | powerful chemoattractant for neutrophils and other granulocytes; strongly associated with promoting tumor angiogenesis, metastasis, and a suppressive myeloid cell-rich tumor microenvironment. |
