# Supplementary material for: Pre−treatment cytokines plus TPSA predict biochemical progression−free survival in prostate cancer metastasis and discriminate metastatic status: a retrospective study
Source: Front Immunol. 2025 Nov 27;16:1686570. doi: 10.3389/fimmu.2025.1686570 (PMC12695854; doi:10.3389/fimmu.2025.1686570)
Supplement: Supplementary file 2 [file Table2.docx]

**Table S2.1 Comparison of Univariate logistic regression analysis using different filling methods**

| ***Statistical Parameter*** | ***Method 1: Linear Regression (MICE)*** | ***Method 2: Predictive Mean Matching (PMM)*** | ***Method 3: Bayesian Linear Regression*** |
| --- | --- | --- | --- |
| Age (y) |  |  |  |
| OR(95%CI) | 1.029（1.002-1.058） | 1.029（1.002-1.058） | 1.029（1.002-1.058） |
| *P* value | 0.038 | 0.038 | 0.038 |
| Smoking |  |  |  |
| OR(95%CI) | 1.041（1.027-1.055） | 1.041（1.027-1.055） | 1.041（1.027-1.055） |
| *P* value | <0.001 | <0.001 | <0.001 |
| TPSA（ng/ml） |  |  |  |
| OR(95%CI) | 1.003（1.002-1.004） | 1.002（1.001-1.004） | 1.003（1.002-1.004） |
| *P* value | <0.001 | <0.001 | <0.001 |
| IL-6（pg/mL） |  |  |  |
| OR(95%CI) | 1.000（1.000-1.000） | 1.000（1.000-1.000） | 1.000（1.000-1.000） |
| *P* value | 0.689 | 0.733 | 0.730 |
| IL-8（pg/mL） |  |  |  |
| OR(95%CI) | 1.007（1.002-1.013） | 1.006（1.001-1.011） | 1.007（1.002-1.013 |
| *P* value | 0.013 | 0.031 | 0.012 |
| IL-10（pg/mL） |  |  |  |
| OR(95%CI) | 0.993（0.971-1.016） | 0.991（0.968-1.015） | 0.996（0.973-1.019） |
| *P* value | 0.544 | 0.478 | 0.744 |
| IL-1β（pg/mL） |  |  |  |
| OR(95%CI) | 0.923（0.780-1.093） | 0.938（0.814-1.081） | 0.933（0.748-1.036） |
| *P* value | 0.354 | 0.374 | 0.363 |
| TNF-α（pg/mL） |  |  |  |
| OR(95%CI) | 1.034（0.961-1.113） | 1.033（0.769-1.386） | 1.034（0.977-1.168） |
| *P* value | 0.365 | 0.337 | 0.369 |

**Table S2.2 Comparison of Multivariate Logistic Regression Analysis with Different Filling Methods**

| ***Statistical Parameter*** | ***Method 1: Linear Regression (MICE)*** | ***Method 2: Predictive Mean Matching (PMM)*** | ***Method 3: Bayesian Linear Regression*** |
| --- | --- | --- | --- |
| Age (y) |  |  |  |
| OR(95%CI) | 1.011（0.981-1.043） | 1.011（0.980-1.042） | 1.012（0.982-1.043） |
| *P* value | 0.473 | 0.495 | 0.443 |
| Smoking |  |  |  |
| OR(95%CI) | 1.038（1.023-1.053） | 1.039（1.024-1.054） | 1.039（1.024-1.054） |
| *P* value | <0.001 | <0.001 | <0.001 |
| TPSA（ng/ml） |  |  |  |
| OR(95%CI) | 1.003（1.001-1.004） | 1.002（1.001-1.003） | 1.002（1.001-1.004） |
| *P* value | <0.001 | <0.001 | <0.001 |
| IL-8（pg/mL） |  |  |  |
| OR(95%CI) | 1.006（1.000-1.012） | 1.006（1.000-1.011） | 1.006（1.001-1.012） |
| *P* value | 0.039 | 0.042 | 0.026 |
