# Supplementary material for: Pre−treatment cytokines plus TPSA predict biochemical progression−free survival in prostate cancer metastasis and discriminate metastatic status: a retrospective study
Source: Front Immunol. 2025 Nov 27;16:1686570. doi: 10.3389/fimmu.2025.1686570 (PMC12695854; doi:10.3389/fimmu.2025.1686570)
Supplement: Supplementary file 3 [file Table3.docx]

**Table S3.** Cytokine members included in 17 combinations.

| *Biomarker Signature Group* | *Team members* |
| --- | --- |
| I | TPSA + IL - 8 |
| II | TPSA + IL - 6 + L - 8 |
| III | TPSA + IL - 8 + L - 10 |
| IV | TPSA + IL - 8 + IL - 1β |
| V | TPSA + IL - 8 + TNF-α |
| VI | IL - 6 + IL - 8 + L - 10 |
| VII | IL - 8 + L - 10 + IL - 1β |
| VIII | IL - 8 + L - 10 + TNF-α |
| IX | TPSA + IL - 6 + IL - 8 + L - 10 |
| X | TPSA + IL - 8 + IL - 10 + IL - 1β |
| XI | TPSA + IL - 8 + IL - 10 + TNF-α |
| XII | IL - 6 + IL - 8 + IL - 10 + IL - 1β |
| XIII | IL - 6 + IL - 8 + IL - 10 + TNF-α |
| XIV | TPSA + IL - 6 + IL - 8 + IL - 10 + IL - 1β |
| XV | TPSA + IL - 6 + IL - 8 + IL - 10 + TNF-α |
| XVI | TPSA + IL - 8 + IL - 10 + IL - 1β + TNF-α |
| XVII | TPSA + IL - 6 + IL - 8 + IL - 10 + IL - 1β + TNF-α |
